# Supplementary material for: Complexome profiling of the Chlamydomonas psb28 mutant reveals TEF5 as an early PSII assembly factor
Source: Plant Cell. 2025 Mar 17;37(6):koaf055. doi: 10.1093/plcell/koaf055 (PMC12164590; doi:10.1093/plcell/koaf055)
Supplement: koaf055_Supplementary_Data [file koaf055_supplementary_data.zip › psb28_interactive.zip!/plots/intensity/qualityPlots/peptides/SHY1_Cre16.g664700.t1.2.html]

### Cre16.g664700.t1.2

Trivial name: SHY1  
SeqCoverage [median]: 4.10  
Mass: 41.23 kDa  
  
 

| Sequence | MissedCleavage | Mass [Da] | wtIntensity | muIntensity | Score |
| --- | --- | --- | --- | --- | --- |
| IVDYQADAR | 0 | 1049.51 | 362.04 | 840.50 | 101.64 |
| SPLEQSR | 0 | 815.41 | 207.97 | 330.92 | 129.47 |
|  |  |  | 570.0 | 1171.4 |  |
